# Research on Improved Depth Belief Network-Based Prediction of Cardiovascular Diseases

**DOI:** 10.1155/2018/8954878

**Published:** 2018-05-09

**Authors:** Peng Lu, Saidi Guo, Hongpo Zhang, Qihang Li, Yuchen Wang, Yingying Wang, Lianxin Qi

**Affiliations:** ^1^School of Electrical Engineering, Zhengzhou University, Zhengzhou 450001, China; ^2^Cooperative Innovation Center of Internet Healthcare, Zhengzhou University, Zhengzhou 450001, China; ^3^Industrial Technology Research Institute, Zhengzhou University, Zhengzhou 450001, China; ^4^State Key Laboratory of Mathematical Engineering and Advanced Computing, Zhengzhou Science and Technology Institute, Zhengzhou 450003, China

## Abstract

Quantitative analysis and prediction can help to reduce the risk of cardiovascular disease. Quantitative prediction based on traditional model has low accuracy. The variance of model prediction based on shallow neural network is larger. In this paper, cardiovascular disease prediction model based on improved deep belief network (DBN) is proposed. Using the reconstruction error, the network depth is determined independently, and unsupervised training and supervised optimization are combined. It ensures the accuracy of model prediction while guaranteeing stability. Thirty experiments were performed independently on the Statlog (Heart) and Heart Disease Database data sets in the UCI database. Experimental results showed that the mean of prediction accuracy was 91.26% and 89.78%, respectively. The variance of prediction accuracy was 5.78 and 4.46, respectively.

## 1. Introduction

Cardiovascular disease has become the most pathogenic disease in our country [1]. The establishment of a prediction model of cardiovascular disease and the quantitative analysis of the risk of disease can effectively reduce the incidence of the disease [2].

In the past few decades, researchers have conducted a lot of research on the computer classification of ECG, such as support vector machines (SVMs), artificial neural networks (ANNs), decision trees, Bayesian networks, support feature machines (SFMs), and regression analysis. Cardiovascular disease prediction model is divided into two categories; one is the traditional prediction model based on probability. For example, in Framingham Heart Study (FHS) [3], the model is characterized by the adoption of a mathematical formula, which has good stability, but its effect is poor and the accuracy is low in the multiclassification and nonlinear complex factors. And the other is based on shallow neural network prediction model of cardiovascular disease. In Munster Heart Study (PROCAM) [4], two neural network models are used: a multilayer perceptron network with one hidden layer (MLP) [5] model and a probabilistic neural network (PNN) [6]. The characteristics of this kind of model are that it can effectively expand the forecasting factor, quickly process the fuzzy data and nonlinear data, and provide high rate of accuracy[7]. However, due to the randomness of initialization of shallow neural network parameters, the prediction results will be much lower than the average accuracy, and the variances of multiple prediction results will be larger.

Recently, deep learning has been widely used in different fields and has made great progress [8–11]. The deep learning model uses multiple samples to extract high-level features and learns hierarchical representations by combining low-level inputs more effectively. The learned features characterize more intrinsic features of the data, avoiding the process of artificial feature design and selection, and have the characteristics of many varieties and high accuracy [12].

This paper takes deep learning as the point of penetration and uses multilayer network architecture to abstract the characteristics of layers and establish a cardiovascular disease prediction model based on deep belief network. At the same time, the prediction model based on deep trust network is improved by using reconstruction error to achieve better prediction.

Overall, the major contributions of this work can be summarized in three aspects. First, we use the deep belief network to build predictive models of cardiovascular disease, skip the morphological feature extraction step, and classify the original ECG data directly, thus solving the problem that the cardiovascular disease prediction model is not robust due to the large difference in waveform characteristics between patients with the same disease. Second, we adopt the best network parameters trained to initialize the neural network, so as to solve the instability problem caused by stochastic initialization. Finally, we use reconstruction error to improve the prediction model which is based on the deep trust network, so that it can independently determine the network depth and achieve better predicted results.

## 2. Related Work

The literature related to this classification application was studied, and it can be seen that a great variety of methods were used, which reached high classification accuracies.

Algorithms for R-peak extraction tend to use wavelet transforms to compute features from the original ECG followed by a fine-tuned threshold-based classifier. Since the accurate estimation of heart rate and heart rate variability can be extracted from the R-peak feature, the specially designed algorithm is usually used for the classification of coarse-grained heart rhythm. Sundar et al. [13] proposed a prototype using data mining techniques, namely, Naïve Bayes and WAC (weighted associative classifier). The recognition rate of 84% and 78% was obtained from weighted associative classifier and Naïve Bayes. Iftikhar et al. [14] present a hybrid approach using a supervised learning model based on a well-known classifier SVM and evolutionary optimization techniques (genetic algorithm (GA) and particle swarm optimization (PSO)). The results have shown considerably improved accuracy of more than 88%.

However, because of the differences in the ECG waveforms of different people and the great differences in ECG waveform characteristics of different diseases, the feature extraction of the waveform is inaccurate. Therefore, these characteristics are not sufficient to distinguish most cardiovascular diseases.

With the rapid development of artificial intelligence, inspired by automatic speech recognition, hidden Markov model with Gauss observation probability distribution has been applied to the beat detection task [15], and the hottest artificial neural network is also used for the task of beat detection [16]. Elsayad proposed an approach which used the learning vector quantization (LVQ) neural network to establish the ECG positive anomaly model and obtained an accuracy of 74.12% [17]. Olaniyi et al. [18] designed a neural network for diagnosis of heart diseases with the heart disease sample obtained from UCI machine learning repository. The system is a multilayer neural network model based on backpropagation training and is simulated on a feed-forward neural network. The recognition of 85% was obtained from testing of the network.

Although the self-learning ability of backpropagation (BP) neural network is strong, the convergence speed is slow, and the result is easily affected by the random initialization of network parameters. In particular, there has been no unified and complete theoretical guidance for the selection of BP neural network structure. Generally, it can only be selected by experience.

The DBN model not only has the self-adaptive ability of the self-adjustment of the general neural network but also avoids the defects of the BP neural network, which is easy to fall into the local minimum. DBN uses a network structure composed of multiple RBM networks, which is more effective for modeling one-dimensional data [19].

## 3. Description of the Proposed Approach

### 3.1. Deep Belief Network

Deep belief network (DBN) is one of the main tools for deep learning, which is based on the restricted Boltzmann machine (RBM) [20], to propose. The structure of RBM includes only the visible layer and the hidden layer; the neurons between two layers are fully connected, and the neurons in the same layer are not connected [21].

**Figure 1:**
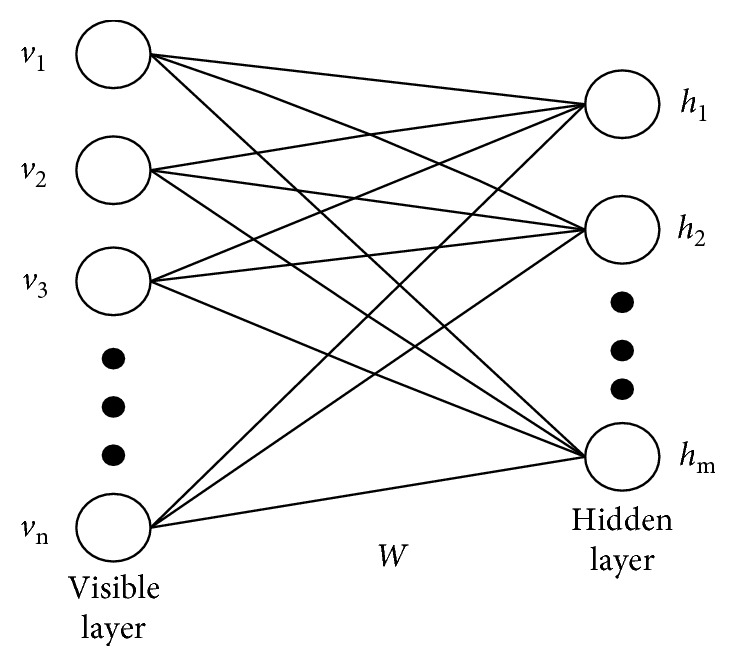
Restricted Boltzmann machine undirected configuration diagram.

In Figure 1, *v*(*v*_1_, *v*_2_,…, *v*_*n*_) represents the visible layer, *v*_*i*_ is the visible unit; *h*(*h*_1_, *h*_2_,…, *h*_*m*_) denotes the hidden layer, *h*_*j*_ is the hidden unit; and *W* is the connection weight matrix between two layers. The data are input from the visible layer, (*v*_1_, *v*_2_,…, *v*_*n*_) represents the feature set of the data, and the hidden layer data are generated by the random initialization of the weight value *w* and the state of each neuron. Due to the disconnection between neurons at the same level, when determining the neuron state, it has the following properties: when the visible cell state is determined, the hidden unit condition is activated independently; otherwise, if the state of the hidden cell is determined, the conditions of the visible units are activated independently.

Given a set of states (*v*, *h*), the energy function of the RBM model can be defined by the following equation:

(1) $$\begin{array}{c} \begin{array}{l} E\left(v,h\right)=-\sum_{i=1}^{n}a_{i}v_{i}-\sum_{j=1}^{m}b_{j}h_{j}-\sum_{i=1}^{n}\sum_{j=1}^{m}v_{i}w_{ij}h_{j},\\ =-a^{T}v-b^{T}h-h^{T}wv, \end{array} \end{array}$$

where *a*=(*a*_1_, *a*_2_,…, *a*_*n*_) denotes the offset vector of the visible unit, *b*=(*b*_1_, *b*_2_,…, *b*_*m*_) denotes the bias vector of the hidden unit, and *v*=(*v*_1_, *v*_2_,…, *v*_*n*_) denotes the state vector of the visible layer, *h*=(*h*_1_, *h*_2_,…, *h*_*m*_) denotes the state vector of the hidden layer, *w*=(*w*_*i*,*j*_) denotes the connection weight matrix, and *w*_*i*,*j*_ denotes the weight of the *i*th visible unit and the *j*th hidden element.

For the state (*v*, *h*), according to (1), the joint probability distribution can be given as follows:

(2) $$\begin{array}{c} \begin{array}{l} P\left(v,h;\theta \right)=\frac{1}{z}e^{-E\left(v,h\right)},\\ z=\sum_{v}\sum_{h}e^{-E\left(v,h\right)}, \end{array} \end{array}$$

where *θ*={*a*, *b*, *w*} is the RMB network parameters and *Z* is called the normalization factor or the partition function.

In practical applications, the probability distribution *p*(*v*) of training data *v* is generally used, that is, the edge probability distribution of *P*=(*v*, *h*, *θ*):

(3) $$\begin{array}{c} P\left(v\right)=\frac{1}{Z}\sum_{h}e^{-E\left(v,h\right)}. \end{array}$$

Similarly, the edge probability distribution *P*=(*h*) of the hidden layer state can be obtained:

(4) $$\begin{array}{c} P\left(h\right)=\frac{1}{Z}\sum_{v}e^{-E\left(v,h\right)}. \end{array}$$

RBM training data are obtained by solving the model optimal parameters in (3), so that the model can better fit the distribution of training data even if the sample reaches the maximum probability in the distribution. Constructing log-likelihood functions:

(5) $$\begin{array}{c} \ln\;P\left(v\right)=\ln\left(\sum_{h}e^{-E\left(v,h\right)}\right)-\ln\left(\sum_{v}\sum_{h}e^{-E\left(v,h\right)}\right). \end{array}$$

The model parameters are respectively solved by the maximum likelihood function method:

(6) $$\begin{array}{c} \begin{array}{l} \frac{\partial \;\ln\;P\left(v\right)}{\partial a}=E_{\mathrm{Pd}}\left[v\right]-E_{\mathrm{Pm}}\left[v\right],\\ \frac{\partial \;\ln\;P\left(v\right)}{\partial b}=E_{\mathrm{Pd}}\left[h\right]-E_{\mathrm{Pm}}\left[h\right],\\ \frac{\partial \;\ln\;P\left(v\right)}{\partial w}=E_{\mathrm{Pd}}\left[vh^{T}\right]-E_{\mathrm{Pm}}\left[vh^{T}\right], \end{array} \end{array}$$

where *E*_Pd_ denotes the expectation of the input conditional probability distribution of training data, and *E*_Pm_ denotes the expectation of the joint probability distribution of the model. The expected computation is done by the Gibbs sampling method, while the computation cost is too large in the computation process of each iteration. Hinton proposed the contrastive divergence (CD) algorithm [21] for the approximate calculation after sampling.

According to the above formula, when the neuron state of the given layer is given, it can be inferred that the activation probability of hidden units is

(7) $$\begin{array}{c} P\left(h_{j}=1\;|\;v\right)=\sigma \left(\sum_{i}v_{i}w_{ij}+b_{j}\right). \end{array}$$

After obtaining the hidden element state matrix, the reconfigurable visible element state probability can be calculated according to the CD algorithm:

(8) $$\begin{array}{c} P\left(v_{i}=1\;\left|\;h\right.\right)=\sigma \left(\sum_{j}h_{j}w_{ij}+a_{i}\right), \end{array}$$

where *σ* is a sigmoid function *σ*(*x*)=11/(1+exp(−*x*)).

The maximum value of the likelihood function is gradually approximated by gradient ascent. The formula of the RBM parameter is updated as follows:

(9) $$\begin{array}{c} \theta^{i+1}=\theta^{i}+\eta \frac{\partial \;\ln\;P\left(v\right)}{\partial \theta }, \end{array}$$

where *η* is the parameter learning rate for the model, and *i* is the current iteration. The parameters *θ* are iteratively updated according to the rules of (9), and the maximum value of the gradient of the likelihood function is reached quickly, which is the optimal parameter.

DBN is composed of a plurality of RBM units connected to the bottom layer of the RBM visible layer as the input layer, the underlying RBM hidden layer of the upper RBM visible layer. The tuning of global training parameters is carried out by the BP neural network.

RBM is a probabilistic neural network that determines the probability generation of DBNs, this is establishing a joint probability distribution between the feature and the lables:

(10) $$\begin{array}{c} P\left(v,h_{1},h_{2},\ldots ,h_{l}\right)=P\left(v\;|\;h_{1}\right)P\left(h_{1}\;|\;h_{2}\right),\ldots ,P\left(h_{l-2}\;|\;h_{l-1}\right)P\left(h_{l-1}\;|\;h_{l}\right), \end{array}$$

where *P*(*h*_*k*_ | *h*_*k*+1_) is the conditional probability distribution of *h*_*k*_ for the given *h*_*k*+1_ state; *P*(*h*_*l*−1_, *h*_*l*_) is the joint probability distribution of *h*_*l*−1_ and *h*_*l*_. *P*(*v*, *h*) is the joint probability distribution of a single RBM. The hidden layer of low-level RBM in DBN is the visual layer of high-level RBM. So (10) is the probability distribution for the whole model.

The use of DBN to establish a deep learning-based cardiovascular disease prediction model is an important entry point to solve the problem of accuracy and stability of prediction models.

### 3.2. Phase 1: Forecasting Model Based on Deep Belief Network

**Figure 2:**
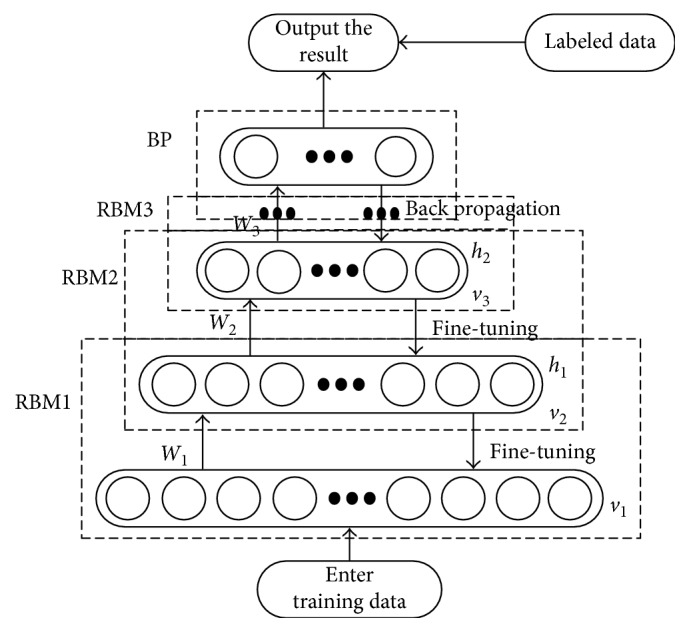
DBNs training flow chart.

The use of deep trust network to establish a cardiovascular disease prediction model is divided into two stages, as shown in Figure 2, respectively, upward training and downward adjustment.

1. Training section: use the greedy layer-by-layer training algorithm to learn the parameters of each layer of RBM *θ*={*a*, *b*, *w*} in turn by unsupervised learning. First, the training data are received by the visible layer of the first layer RBM, and the state *v*_1_ is generated. The hidden state *h*_1_ is generated upwards by the initialized weight matrix *w*_1_, and the visible layer state *v*_1_′ is reconstructed by *h*_1_. Generating new hidden units, the new layer is generated by *w*_1_ remapping to the hidden unit *h*_1_′. The parameters are updated using the CD algorithm until the reconstruction error is least, that is, to complete the first layer RBM training. Stacked RBMs are trained layer by layer according to greedy learning rules, each layer maps different feature spaces The topmost RBM bidirectional connections make up the associative memory layer, which can be associated with the optimal parameters of memory layers. By unsupervised learning, the DBNs gains a prior knowledge, obtains more abstract features at the top level, and better reflects the real structure information of the training data. Stacked RBM pretraining input is as follows: training data *x*, DBN; and output is as follows: unsupervised DBN.
2. Tuning section: taking the pre-trained parameters of the network as initial values, the labeled samples are used to supervise the DBN model and the top-down reverse propagation error of the network is used as the standard to further optimize the RBM parameters of various layers. The initial value of BP network is the high abstract feature set obtained by the pretraining of DBN, which solves the problem of falling into local optimum and overfitting caused by random initialization of the traditional neural network. The parameters are finetuned based on the BP algorithm, and the input is the parameters of each layer of the DBN pretraining and the output vector of the top RBM; the output is the DBN after finetuning the parameters.

Through the above steps, a globally optimal DBN model is constructed and fully trained. To sum up the above learning phase, a complete DBN model is established, and the input is as follows: number of DBN structure layers, training samples; output is as follows: fully trained DBN.

Cardiovascular disease training samples without label values were entered into the visible layer of the bottom RBM without any characteristics of supervised learning data. The top RBM will learn the optimal characteristic parameters as the initial value of the neural network solves the defects caused by random initialization and improves the stability of the model prediction.

### 3.3. Phase 2: Improved Deep Belief Network Forecasting Model

The more complex the network structure of DBN, the stronger the ability to solve complex problems. Simultaneously, the higher the number of network layers, the harder the training will be, the greater the training error accumulates, and the lower the correctness of the model [22]. In application, in order to establish suitable DBN structure for specific tasks, due to lack of corresponding theoretical support and effective training mode, the depth of network and the number of hidden units need to be set by experience, which leads to the deviation in the modeling process and the high cost [23].

Aiming at the problem of determining the number of layers of DBN, based on the reconstruction error of each RBM training, this paper improves the prediction model of deep trust network and establishes a DBN which can automatically select the network depth to improve the automatic analysis ability of the cardiovascular disease prediction model. Specific methods are as follows.

In each RBM, the input data of the visible layer are reconstructed and mapped to the hidden layer again, and the reconstruction error is calculated based on the difference between the reconstructed output data and the initial training data.

(11) $$\begin{array}{c} R_{\text{error}}=\frac{\sum_{i=1}^{n}\sum_{j=1}^{m}p_{ij}-x_{ij}}{nmp_{x}}, \end{array}$$

where *R*_error_ denotes the reconstruction error, *n* denotes the number of training samples, *m* denotes the number of features in each group of samples, *P*_*ij*_ denotes the reconstructed value of RBM training sample per layer, *x*_*ij*_ denotes the true value of the training sample, and *P*_*x*_ denotes the calculation of the number of values.

In order to prevent the training data from overfitting or reconstructing large deviation of the data and at the same time to balance the training cost of the network model, when the difference between the two reconstruction errors is less than the present value, the depth accumulation is stopped.

(12) $$\begin{array}{c} \begin{array}{l} L=N_{\text{RBM}}+1,\\ \left|R_{\text{error}}\left(k-1\right)-R_{\text{error}}\left(k\right)\right|>\varepsilon ,\\ L=N_{\text{RBM}},\\ \left|R_{\text{error}}\left(k-1\right)-R_{\text{error}}\left(k\right)\right|<\varepsilon , \end{array} \end{array}$$

where *L* denotes the hidden layer number of DBN, *R*_error_ (*k*) denotes *R*_error_ of current layer, and *ε* denotes the default value. The selection of the preset value is one of the keys to determining the accuracy of the model. The value of the default value *ε* is too large, which can cause inaccurately finding the optimal number of network layers. If the value is too small, the number of layers in the deep neural network may be too large and the calculation amount is too large. For the number of cardiovascular disease prediction model parameters and the performance of laboratory equipment, we determined that *ε* ∈ [0.01, 0.05]. Compared with many experimental results, when *ε*=0.03, the prediction model can determine the network depth independently.

**Figure 3:**
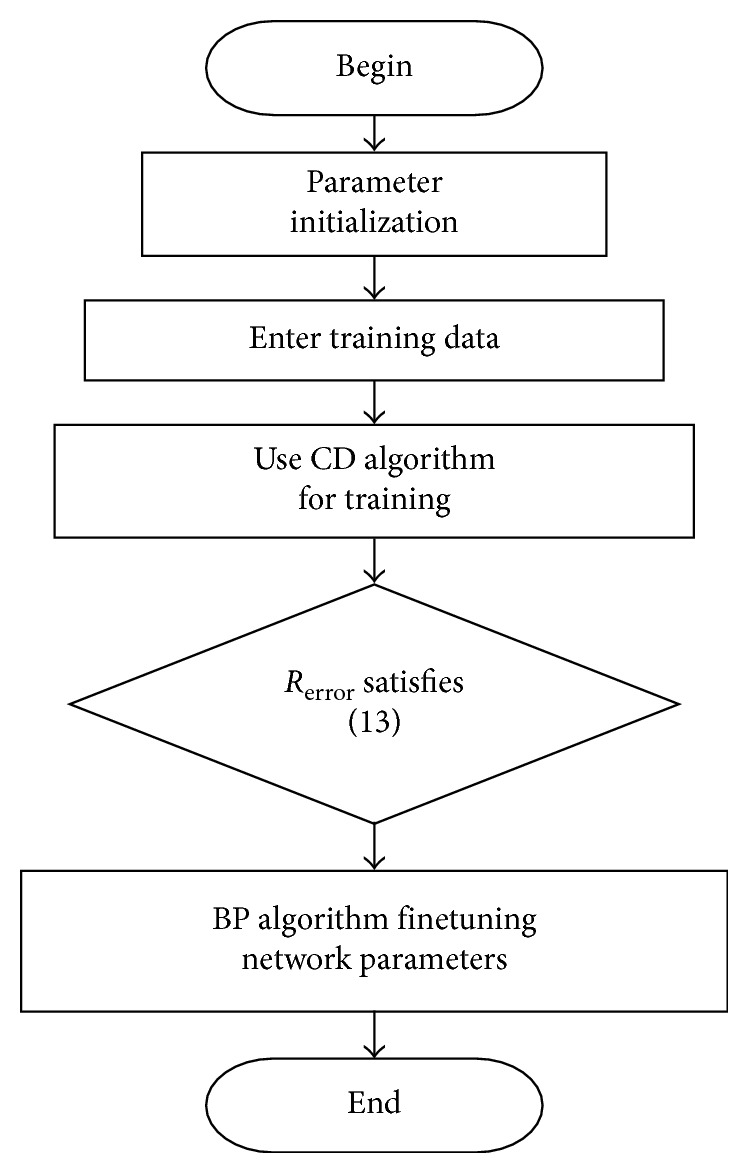
Calculation flowchart of DBNs depth.

In the pretraining phase of the unsupervised, when it reaches the number of layers of target value, the top-level trained output is used as input of the BP algorithm and the reverse fine-tuning parameters are started. The process of building a network relies on *R*_error_, as shown in Figure 3.

*R*_error_ is positively related to the network energy *E*(*v*, *h*), and this coupling characteristic also proves feasibility of DBN depth with the reconfiguration error as the standard. It is proved as follows.

Let *P* be the calculated value, and *X* be the actual label value, then *P*=*P*(*v*) and *X*=*P*(*v*_1_); according to the conditional probability formula, there is

(13) $$\begin{array}{c} P=P\left(v\right)=P\left(v_{1}\right)P\left(h\;|\;v_{1}\right)P\left(v\;|\;h\right). \end{array}$$

According to the total probability formula, there is

(14) $$\begin{array}{c} P\left(v\;|\;h\right)=\frac{P\left(v,h\right)}{P\left(h\right)}. \end{array}$$

According to (14), to rewrite (13), there is

(15) $$\begin{array}{c} \begin{array}{l} P=P\left(v_{1}\right)\frac{P\left(v_{1},h\right)}{P\left(v_{1}\right)}\frac{P\left(v,h\right)}{P\left(h\right)},\\ \;=P\left(v_{1},h\right)\frac{P\left(v,h\right)}{P\left(h\right)}. \end{array} \end{array}$$

According to (14) again, there is

(16) $$\begin{array}{c} \begin{array}{l} P=P\left(v_{1}\;|\;h\right)P\left(h\right)\frac{P\left(v,h\right)}{P\left(h\right)},\\ \;=P\left(v_{1}\;|\;h\right)P\left(v,h\right). \end{array} \end{array}$$

Substituting the above formula in (11) to reconstruct the error:

(17) $$\begin{array}{c} \begin{array}{l} R_{\text{error}}=\frac{\sum_{i=1}^{n}\sum_{j=1}^{m}\left(p_{ij}-x_{ij}\right)}{nmp_{x}},\\ =P-X,\\ =P\left(v_{1}\;|\;h\right)P\left(v,h\right)-P\left(v_{1}\right),\\ =P\left(v_{1}\right)\left[P\left(v,h\right)-1\right]. \end{array} \end{array}$$

As the energy of the neural network is proportional to the probability distribution, that is, *P*(*v*, *h*)⁡*∞*⁡*E*(*v*, *h*), there is

(18) $$\begin{array}{c} R_{\text{error}}\infty P\left(v,h\right)\infty E\left(v,h\right). \end{array}$$

Equation (18) shows that there is a coupling relationship between *R*_error_ and network mechanism, and it is reasonable to rely on reconstruction error to determine the network depth of DBN autonomously. The number of neurons in each layer also has an impact on the network. At present, there is a lack of a clear theory to prove that the appropriate number of cells is set and the improvement is achieved. The DBN structure focuses on the ability to determine the depth of a network, and the number of neurons in each layer is fixed.

## 4. Experiment Analysis

### 4.1. Database Description

Experimental data select the Statlog (Heart) data set and the Heart Disease Database data set for the UCI Machine Learning Library. The Statlog (Heart) data set contains 270 sets of instances and the Heart Disease Database data set contains 820 sets of instances. The properties of both data sets contain continuous, two-category, ordered multiclass, and unordered multiclass variables. As shown in Table 1, select the same 13 attributes and 1 classified label values in two data for experiments.

The physical meaning, data unit, and order of magnitude of each attribute in the selected data set are different and need to be normalized before the experiment. Text-based data are directly converted to numeric data. The reference standard for medicine is the data attribute of the hierarchical classification structure. The normalized assignment is the corresponding discrete arithmetic progression or geometric progression. For the data attributes of the range type, we proposed improved min-max normalization due to the existence of data imbalances: take the average of the first *k* large values of the feature term as the maximum value, and take the average of the first *k* small values as the minimum value. The feature item is normalized to the interval (0, 1) as min-max.

In the two data sets, 70% of the instances are selected as training samples, and the remaining instances are test samples. The data set is divided into two mutually exclusive collections, and the consistency of data distribution is maintained as much as possible.

### 4.2. Improved DBN Model Network Depth Analysis Experiment

#### 4.2.1. Improved DBNs Model Experiment

Using training data, improved DBN models are built and tested with test data. Inputs are as follows: training sample risk factor data {*x*}, training sample tag value {*y*}, and testing samples {*x*′, *y*′}; the output is the forecast results. The steps are as follows:

1. Set the initial value of the network, the learning rate is set to 1, the initial error is 0, the setting error of the reconstruction error *ε* is set to 0.03, the maximum training period of each RBM is set to 10 times. The weight (w), the visible layer bias (a), and the hidden layer offset (b) are all randomly generated values that are smaller, and the training batch is set to 100.
2. The training data {*x*} with the label value removed is input as the first layer network and the unsupervised pretraining phase is started. The number of neurons in the input layer automatically takes the value of the sample feature dimension, that is, 13 risk factors in the data set. Perform the following steps using Gibbs sampling and CD algorithms, as shown in Table 2.

Update the parameters and calculate the error and repeat the above steps until the end conditions are met. In this case, the first layer of RBM is trained, and the principle of reconfiguring the error method to determine the depth of the network is used to calculate whether the condition is met; if it is satisfied, it stops; if it is not, *h*_1_′ is used as the input for the next layer of training.

(3) Use step (2) to determine the final depth of the network, and remember the optimal parameters of each layer. The trained DBN structures and the parameters are passed to the BP network to build the same depth of backpropagation network.
(4) The top RBM output for the BP network input, while inputting the training data label value, began to monitor the tuning phase and further adjust the parameters of the DBN layers.
(5) Put the unlabeled test data into the constructed improved DBN, and compare the value of the label value of the network to the true label value to calculate the prediction accuracy.
(6) The algorithm ends.

#### 4.2.2. Standard DBNs Model Experiment

In order to improve the correctness of the network depth determined by DBN autonomously, a standard DBN is established and the optimal network layer number is determined by experiment. The optimal number of cells in each layer is experimentally selected according to

(19) $$\begin{array}{c} N=\left\lceil \sqrt{mn}\right\rceil +k, \end{array}$$

where *m* denotes the dimension of the input data, that is, the number of CVD risk factors; *n* denotes the number of output layer units and CVD predicts the probability as the output, that is, *n*=1; *N* is the number of hidden units; ⌈⌉ is the uplift symbol; and *k* is an integer between [1, 5], which is used to increase the interval of units selection and avoid blind selection.

#### 4.2.3. Experimental Results and Analysis

**Figure 4:**
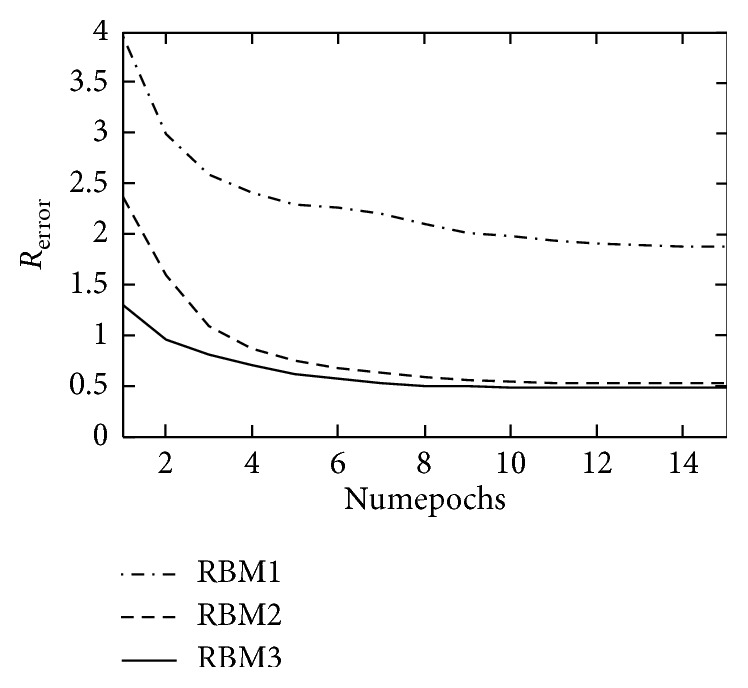
The *R*_error_ curve of each layer RBM on Statlog (Heart).

**Figure 5:**
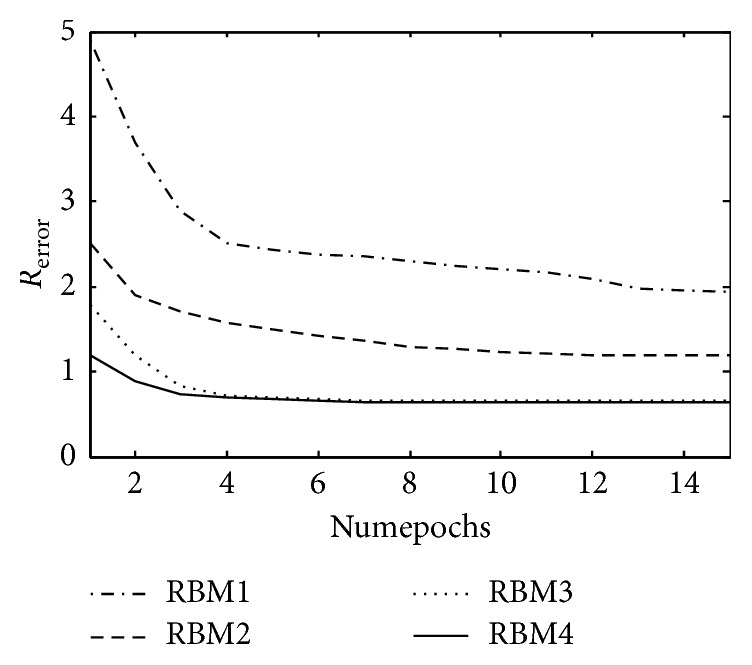
The *R*_error_ curve of each layer RBM on Heart Disease Database.

The improved DBN prediction model was tested in two data sets and stopped increasing when Statlog (Heart) was added to the third layer, with a depth of 4; the Heart Disease Database stopped increasing when it increased to the fourth level with a model depth of 5. The *R*_error_ curve in the RBM computing process of each layer is shown in Figures 4 and 5.

**Figure 6:**
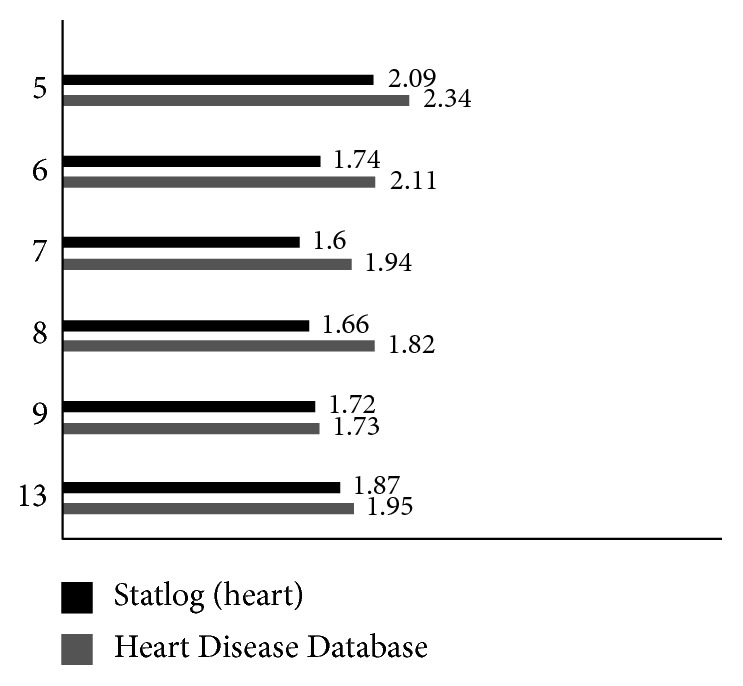
Reconstruction error of RBM1 with different number of hidden units.

In order to improve the performance of DBN, a standard DBN model with the same structure was established, that is, a 4-layer neural network was established for Statlog (Heart) and a 5-layer neural network was established for the Heart Disease Database. The number of network units per layer was based on (19), and the best number of units is selected by the experimental method. The number of input layer units is equal to 13 feature latitudes of the data set, that is, *m*=13; the network output is a label probability obtained by regression calculation, that is, *n*=1; and the number of second layer units ranges from 5 to 9 experiments to select the smallest reconstruction error as the optimal unit number, the number of units under the reconstruction error shown in Figure 6.

As shown in Figure 6, the *R*_error_ of RBM1 in the Statlog (Heart) data set is the smallest at the 7th implicit unit, and the number of units is determined to be 7. The Heart Disease Database has the smallest *R*_error_ at the 9th implicit unit, and the number of units is determined to be 9. Similarly, the DBN structure finally determined according to the above method is Statlog (Heart): 4-layer network, the number of units of per layer is 13-7-6-4; Heart Disease Database: 5-layer network, the number of units of per layer is 13-9-8-5-4.

To further improve the correctness of the network depth determined by DBN, we increase the hidden layer number of the standard DBN model in Figure 6.

Reconstruction error of RBM1 with different numbers of hidden units turns and judges the correctness of the test data. To ensure that the number of layers is the only independent variable, the number of units in each layer is the same as that of the improved DBN model. The results are shown in Table 3.

Analysis of Table 3 shows that increasing the network hierarchy reduces *R*_error_ and training time will increase. The accuracy of the test data was maximized for Statlog (Heart) at depth 4, maximum for the Heart Disease Database at depth 5, and in line with the improved network depth that DBN automatically determines; it further proves that the prediction model of cardiovascular diseases based on improved DBN has better performance.

Table 4 presents the overall results of the proposed Statlog (Heart) data set evaluation using the UCI Machine Learning Library for the proposed improved DBN prediction model and other different hybridization and nonhybrid techniques for cardiac classification and identification of relevant risk factors.

From the comparison of the tables, we can see that the traditional feature extraction algorithm is more specific to a specific data set. Based on the experimental accuracy rate, a special manually set feature combination is used. This method is to dig out the characteristics of the data set itself, not the essential characteristics of ECG data; the generalization ability of the method is weak, the portability is poor, and the accuracy is relatively poor.

The traditional classification model based on probability uses a combination of multiple feature extraction methods. However, the deep learning method can learn a kind of deep-level nonlinear network structure and can effectively obtain the deep-level essential feature representation of ECG from the sample. The effectiveness of the model based on deep learning is better than that of the traditional classification models based on probability and shallow neural networks.

This paper constructs a deep confidence network which can independently determine the network structure. The performance of the model is evaluated on two data sets, and the highest accuracy is achieved. The algorithm has strong generalization ability, and it can fully tap the deep-level characteristics of ECG and achieve an accurate and stable automatic classification of cardiovascular diseases in complex individuals and complex environments. The performance of heart disease classification is superior to other technologies.

## 5. Conclusion

For these issues, the probabilistic-based predictive model cannot integrate multiclass and nonlinear factors, and the stability of shallow neural network is poor. A prediction model based on deep learning is proposed and improved to enable it to independently determine the network parameters. The proposed prediction model was validated with the Statlog (Heart) data set and the Heart Disease data set, which proves that the prediction model has high accuracy and good stability.

Our further research is to apply the prediction model based on improved depth learning to actual cardiovascular disease predictions. By analyzing the prediction results in detail, we can quantify the proportion of each risk factor to the risk of cardiovascular disease and provide personalized advice to reduce the risk of cardiovascular disease.

## Figures and Tables

**Table 1:** Data set attributes.

Features	Description	Data types	Normalization	Value
Age	Age	Continuous data	Min-max scaling	16–80; 0∼1

Sex	Gender	Text-based data	Direct mapping	0: female
				1: male

Cp	Chest pain type	Text-based data	Direct mapping	0: typical angina
				1: typical type angina
				2: nonangina pain
				3: asymptomatic

Trestbps	Trest blood pressure	Range data	Improved min-max scaling	MmHg on admission to the hospital

Chol	Serum cholesterol	Range data	Improved min-max scaling	(mg/dl)

Fbs	Fasting blood sugar	Hierarchical data	Hierarchical mapping	0: <120 mg/dl
				1: >120 mg/dl

Restecg	Resting electrographic results	Text-based data	Direct mapping	0: normal
				1: having ST-T wave abnormality
				2: showing probable or definite left ventricular hypertrophy

Thalach	Maximum heart rate achieved	Range data	Improved min-max scaling	—

Exang	Exercise-induced angina	Text-based data	Direct mapping	0 = no
				1 = yes

Oldpeak	ST depression induced by exercise relative to rest	Range data	Improved min-max scaling	

Slope	Slope of the peak exercise ST segment	Text-based data	Direct mapping	0: unsloping
				1: flat
				2: downsloping

Ca	Number of major vessels colored by fluoroscopy	Text-based data	Direct mapping	0–3

Thal		Text-based data	Direct mapping	0: normal
	1: fixed defect		
	2: reversible defect		

Num	Predicted attribute	—	—	0, 1, 2, 3, 4

**Table 2:** Algorithm implementation steps.

*h*_1_=sigm(*v*_1_^*T*^*w*_1_+*b*)	Use the input data to construct a hidden unit state
*v*_1_′=sigm(*h*_1_^*T*^*w*_1_+*a*)	Reconstruct the input using the hidden layer structure
*h*_1_′=sigm(*v*_1_^′*T*^*w*_1_+*b*)	Construct the hidden layer with the reconstructed input again

**Table 3:** DBNs test results at different network depths.

Data set	Network depth	*R*_error_	Accuracy (%)	Runtime (s)
Statlog (Heart)	2	1.8700	81.43	17.30
	3	0.5189	85.71	19.90
	4	0.4912	91.26	22.70
	5	0.4306	87.14	25.20
	6	0.3877	82.58	29.50

Heart Disease Database	2	1.9450	80.40	21.40
	3	1.1931	83.20	22.90
	4	0.6594	85.60	25.20
	5	0.6372	89.78	27.80
	6	0.5765	87.20	31.50

**Table 4:** Comparison of classification results of different techniques on the Statlog (Heart) data set.

Classification algorithms	Accuracy (%)
SVM linear [14]	86.62
SFM [24]	83.31
OCSFM [24]	86.73
Naïve Bayes [13]	78.93
WAC [13]	84.00
ANN [14]	86.04
PSO-SVM-SMO-RBF [14]	88.24
Naïve Bayes [25]	82.31
Decision tree [25]	84.35
LVQNN [17]	74.12
BPNN [18]	85.00
Improved DBN	91.26
